# Knowledge, perceptions, and attitudes by residents in Punjab and Khyber Pakhtunkhwa, Pakistan in connection with bats

**DOI:** 10.1186/s13002-022-00541-9

**Published:** 2022-06-04

**Authors:** Attaullah, Shahzad Ali, Arshad Javid, Muhammad Imran, Tahir Mehmood Khan, Kendra Phelps, Kevin J. Olival

**Affiliations:** 1 Wildlife Epidemiology and Molecular Microbiology Laboratory (One Health Research Group), Discipline of Zoology, Department of Wildlife and Ecology, University of Veterinary and Animal Sciences, Lahore, Ravi Campus, Pattoki, Pakistan; 2 Department of Wildlife and Ecology, University of Veterinary and Animal Sciences, Lahore, Ravi Campus, Pattoki, Pakistan; 3 Institute of Biochemistry and Biotechnology, University of Veterinary and Animal Sciences, Lahore, Pakistan; 4 Institute of Pharmaceutical Science, University of Veterinary and Animal Sciences, Lahore, Pakistan; 5 EcoHealth Alliance, New York, USA

**Keywords:** Community knowledge, Fruit bats, Bats-human conflicts, Bats conservation, Pakistan

## Abstract

**Background:**

Fruit bats play an important role in pollination and seed dispersal, and their conservation is important to maintain the productivity of some crops and natural ecosystems. The objective of this study was to investigate the knowledge, attitudes, and perception of fruit bats by orchard farmers and agricultural communities in Pakistan.

**Methods:**

The present survey was conducted in two districts (i.e. Sheikhupura and Malakand districts) within Punjab and Khyber Pakhtunkhwa provinces based on the higher number of fruit growing areas and bat roosting sites. A total of 200 (100 per district) close-ended questionnaires with 53 questions were administered to randomly selected respondents within the selected communities associated with fruit orchards, including orchard owners, laborers, and members of the surrounding community. Each questionnaire was divided into seven sections (i.e., demographic information, environmental and public health effects of bats, knowledge about bats, perception and control of bats, non-lethal methods adopted to control bats, and different myths about bats).

**Results:**

A majority of respondents (59%, *n* = 118) mis-classified bats as birds instead of mammals despite more than 84% reporting that they have observed bats. Nearly 71.5% of orchard farmers perceived that their fruits are contaminated by bats during consumption, and a majority believe that bats destroy orchards (62.5%) and are responsible for spreading disease. Mythology about bats was ambiguous, as 49% of those surveyed did not perceived bats to bring good luck (49%), and 50% did not perceived them to be bad omens either. Most respondents have never killed a bat (68%) nor would they kill a bat if given the opportunity (95%). Regarding the control of bats, the greatest percentage of respondents strongly disagree with shooting bats (36%) and strongly agree with leaving bats alone (42.5%).

**Conclusions:**

This study provides a better understanding of the sociodemographic factors associated with knowledge, attitude and perception of bats from fruit orchard owners, labourers and local people. We recommend educational interventions for targeted groups in the community, highlighting the ecosystem services and importance of bat conservation to improve people’s current knowledge regarding the role of bats and reduce direct persecution against bats.

## Background

Agriculture is the largest economic sector of Pakistan and fruit farming is one of the sub-sectors which plays an important role in the development rural communities. Approximately 45% of the labor in the country is directly or indirectly dependent on agriculture [1, 2]. Mangoes, bananas, guava, dates, and citrus are key fruits found in the tropical and sub-tropical climates of Pakistan. While apple, guava, grapes, orange, pear, persimmon, banana, and peach are the main commercial fruits of Pakistan with a total cultivation area of 1.3 million hectares [2, 3], the success and spread of these fruit trees depend largely on pollination, pest control, and seed dispersal by insects, birds and mammals such as bats.

Bats belong to the Order Chiroptera and include more than 1,400 species widely distributed across all continents except Antarctica [4]. There are fifty recognized species of bats in Pakistan which belong to twenty-six genera and eight families [5]. Some bat species roost and forage in fruit-growing regions, and provide critical ecological and economical services, such as seed dispersion, pollination, and pest control [6, 7]. Specifically, bats pollinate crops of socio‐economic importance, such as banana, durian, and mango [8]. Insectivorous and frugivorous species play roles in pollination and seed dispersal of wild plants and with their faeces they add nutrients to the soil in which the plants grow [9].

However, due to lack of knowledge most orchard farmers in Pakistan believe that all the bats are fruit consumers and are considered vermin [10]. Farmers in other regions of Asia also consider bats as agricultural pests [11, 12]. Negative attitudes of farmers towards bats and the hunting of bats for food and medicinal purposes threatens the long-term viability of local bat populations [12–14]. In addition to the above-mentioned threats, deforestation, global warming, roost site disturbance, disease, and over-exploitation also threaten bat populations [15]. Such threats to bat populations are further exacerbated by the negative perception of bats by the general public as bats are perceived to be carriers of zoonotic diseases [16, 17]. Another common misunderstanding is that bats feed on blood, but in fact only three species consume blood and are restricted to Central and South America [18, 19]. The present study aimed to investigate the knowledge, attitudes, and perception of fruit bats by orchard farmers and surrounding communities in two districts within Punjab and Khyber Pakhtunkhwa provinces.

## Methods

### Study area

This study was conducted in two provinces, Punjab and Khyber Pakhtunkhwa (KPK), in Pakistan from January to October 2019. We selected one district of Punjab (i.e. Sheikhupura) and one district of KPK (i.e. Malakand). Areas were selected based on the higher number of fruit growing areas and the bats roosting sites. The main fruit orchard found in these districts were persimmon, citrus, loquat, litchi, orange, pears, plum, guava, mango and papaya. Most of the fruit bats were attracted to fruit trees which are evergreen and may provide suitable foraging sites. Some common fruit bats species found in these study sites were Indian fly fox (*Pteropus medius*), Egyptian fruit bat (*Rousettus aegyptiacus*), Fulvous fruit bat (*Rousettus leschenaultia*), and the short-nosed fruit bat (*Cynopterus sphinx*). Insectivorous' bats observed at the study sites include the common pipistrelle (*Pipistrellus pipistrellus*), the greater mouse-tailed bats (*Rhinopoma microphyllum*) and Asiatic lesser yellow house bat *(Scotophilus kuhlii*). All of these bats species roosts were found in old buildings, on trees and caves [5, 20]. These bats were identified by bat ecologists of EcoHealth Alliance, New York, USA and Department of Wildlife and Ecology, University of Veterinary and Animal Science, Lahore, Pakistan by using standard keys [5].

### Questionnaire design

We developed a questionnaire to find out conflicts between bats and fruit orchard owners, labourers working in orchards and the local people around the orchards of and Punjab and KPK [19, 21].

A closed-ended questionnaire was prepared in English, however it was translated into Urdu and Pashto to cater to local use. The original questionnaire consisted of 52 questions. The questions were simple and easily understood by any local people. Questions were not only simple but also provided an overall estimation of their perception and knowledge about bats. Authors of this study reviewed and revised the questionnaire.

We divided the questionnaire into seven sections: the first section of the questionnaire consisted of seven-question general/demographic information including district, gender, age, marital status, location, education level and sector of employment. In the second section we asked sixteen questions to assess local knowledge about bats, including basic bat taxonomy (i.e. are they the same as birds), whether populations of bats have been observed to be decreasing, if bats should be conserved, and if bats have negative vs. positive impacts on agriculture. In the third section twelve statements were added regarding environmental and public health effects of bats such as awareness regarding potential of bats to contaminate water, zoonotic diseases hosted by bats, bats impact on fruit damage, and efficacy of bat guano for fertilizer**.** The fourth section was about perception and attitudes towards bats. This part had seven statements including feeling tense around bats, friendliness towards bats, and whether killing of bats is a good thing. In the fifth section we asked four questions about perception and control of bats such as if bats should be shot, trees should be cut down to get rid of the bats, poisons should be used, or if these bats should be left alone. For each of these statements, four options were presented, e.g. strongly agree, agree, disagree strongly disagree, and don’t know. In the sixth section of the questionnaire we asked four questions about non-lethal methods adopted for control of bats to prevent fruit damage, e.g. light, net, sound, and noxious smell in which the respondents indicate whether they agree to disagree, don’t know. The last section of the questionnaire was about myths about bats, e.g. symbols of bad omen and cure of disease, and whether bats should be used as medicine with the option of agree, strongly agree, disagree, and don’t know.

### Data collection

This study was conducted from February to June 2019. Total 200 (100 per district) questionnaires were circulated among the people including the owners of the orchard, tenants, contractors, labor working in orchards and the local people around the orchards. A team of two peoples (one Pashtun and one Punjabi) with good knowledge of the local language and area were recruited to administer the surveys. Data were collected by face-to-face interviews from randomly selected participants. Each participant was briefed about the purpose of this survey and its goal of understanding bats-human interactions in the study area.

### Statistical analysis

Data were analyzed by SPSS version 25 to generate summary statistics and run chi square test to indicate significant difference (*p*-value < 0.05) among different responses of each question/variable.

## Results

### Demographic information

**Fig. 1:**
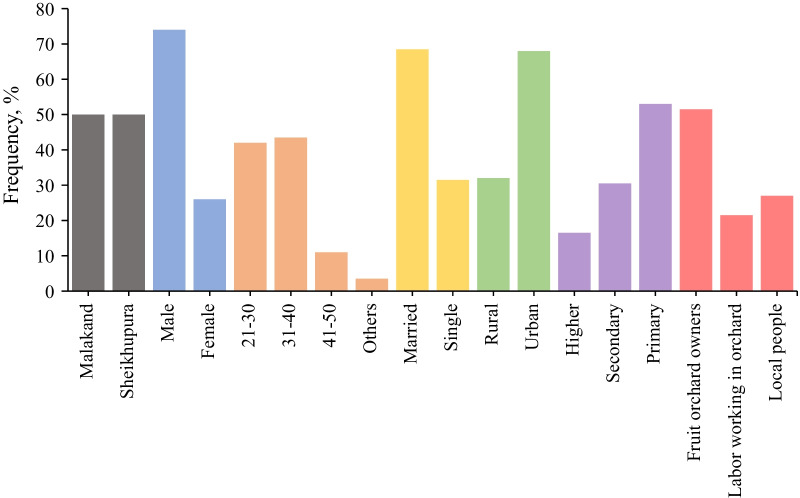
Socio-demographic variables and frequency of different characteristics of the respondents (*n* = 200)

A total of 200 respondents were included in this survey. Regarding the demographic information, a total of 7 questions were covered (Fig. 1). All respondents (*n* = 200) were Muslims and belonged to Malakand and Sheikhupura (*n* = 100; 50% of each region). The number of male respondents (*n* = 148; 74%) was higher than that of the female respondents (*n* = 52; 26%). Among different age groups, respondents of 31–40 years of age group (*n* = 87; 43.5%) were highest, followed by the age groups of the 21–30 year-olds (*n* = 84; 42%), 41–50 year-olds (*n* = 22; 11%), and others (*n* = 7; 3.5%). Most of the respondents were married (*n* = 137; 68.5%), while 31.5% (*n* = 63) were single. Among the locations, the number of urban respondents (*n* = 136; 68%) was higher than that of the rural respondents (*n* = 64; 32%). Most of the respondents had primary level of education (*n* = 106; 53%), whereas, 30.5% (*n* = 61) were of the secondary level, and 16.5% (*n* = 33) were of the university level. Among different sectors, the number of respondents was highest in the sector of fruit orchard owners (*n* = 103; 51.5%), followed by the labourers working in the orchard sector (*n* = 54; 27%), and the local people (*n* = 43; 21.5%).

### Basic knowledge about bats

All respondents (*n* = 200) were asked questions regarding basic knowledge about bats (Table 1). Most of the respondents claimed that the bats are birds (*n* = 118; 59%), and that they have seen bats (*n* = 168; 84%). A greater number of respondents having fruit orchards in their area (*n* = 189; 94.5%) claimed that the fruits are contaminated or damaged (*n* = 142; 71%) by bats during consumption (*n* = 140; 70%), and they wash the fruits before eating (*n* = 195; 97.5%). Among respondents, the majority claimed no direct contact with bats, e.g. capturing of bats with unprotected hands (*n* = 117; 58.5%)—although interestingly 41.5% did report some direct contact with bats. Most participants did not witness bat movement directly (*n* = 106; 53%), e.g. regular observations of bats in the evening from roosts (*n* = 113; 56.5%).

**Table 1:** Respondents count (*n*) and frequency (%) of the responses for questions regarding basic knowledge about bats

Questions/Variables	Do not know *n* (%)	No *n* (%)	Yes *n* (%)	*χ*^2^	*P*-value
Bats are birds?	5 (2.5)	77 (38.5)	118 (59)	35.7	< 0.01
Have you ever seen bats?	0 (0)	32 (16)	168 (84)	9.52	0.02
Fruits are the common food consumed by bats	11 (5.5)	49 (24.5)	140 (70)	16.39	0.01
Fruits are contaminated/damaged by bats	8 (4)	50 (25)	142 (71)	9.45	0.15
Do you wash fruits before consumption?	0 (0)	5 (2.5)	195 (97.5)	15.38	< 0.01
Do you have any fruits orchards in your area?	0 (0)	11 (5.5)	189 (94.5)	34.92	< 0.01
Direct contact with bats	0 (0)	117 (58.5)	83 (41.5)	31.53	< 0.01
Have you seen roost of bats?	2 (1)	85 (42.5)	113 (56.5)	9.38	0.15
Have you observed the movement of bats from their roosts every evening	11 (5.5)	83 (41.5)	106 (53)	24.79	< 0.01
Population of bats decrease over the last 3 years	43 (21.5)	77 (38.5)	80 (40)	56.26	< 0.01
Should bats be conserved?	32 (16)	81 (40.5)	87 (43.5)	36.06	< 0.01
Have any positive effects on agriculture?	29 (14.5)	82 (41)	89 (44.5)	35.78	< 0.01
Bats destroy our orchards	9 (4.5)	66 (33)	125 (62.5)	25.62	< 0.01
Killing of bats is good thing	3 (1.5)	114 (57)	83 (41.5)	30.18	< 0.01
Bats are spreading infectious diseases	27 (13.5)	78 (39)	95 (47.5)	41.61	< 0.01
Do people eat bats?	27 (13.5)	173 (86.5)	0 (0)	4.58	0.205

Knowledge about the population trends of bats were ambiguous, 38.5% (*n* = 77) claimed an increase in local populations over the last 3 years, and 40% (*n* = 80) reported a decrease, whereas, 21.5% (*n* = 43) were unclear about the increase or decrease of the bat population. Among respondents, 43.5% (*n* = 87) claimed that bats should be protected, whereas, 40.5% (*n* = 81) believed that the bats should not be protected. Most of the participants responded that bats destroy fruit in orchards (*n* = 125; 62.5%), but, 44.5% (*n* = 89) believed that the bats have positive effects on agriculture, and killing of bats is not a good practice (*n* = 114; 57%). Nearly half of the respondents claimed that bats spread infectious diseases (*n* = 95; 47.5%), and a majority believe that eating bats is not a good practice (*n* = 173; 86.5%).

### Environment and public health effects

Respondents were asked questions regarding the environment and public health effects of bats (Table 2). Most of the respondents claimed that the bats make a noise (*n* = 106; 53%), destroy crops and fruits in the environment (*n* = 92; 46%), contaminate the water (*n* = 108; 54%) and spread zoonotic diseases (*n* = 90; 45%). A greater number of respondents were aware that bats feed on crop pests (*n* = 94; 47%) and help with insect control (*n* = 119; 59.5%). The majority of the respondents claimed that the bats are helpful in tree planting (*n* = 88; 44%) by dispersing seed (*n* = 112; 56%) in the environment and their droppings are good fertilizer (*n* = 97; 48.5%). Among the respondents, a higher number claimed that bats inflict economical losses (*n* = 96; 48%) by dropping fruits from trees (*n* = 127; 63.5%). Overall, more respondents (*n* = 88, 41%) believed that bats provided benefits to people, although 41% of respondents did not agree with this statement.

**Table 2:** Respondents count (*n*) and frequency (%) of the responses for questions regarding the environment and public health effects of bats

Parameters	Do not know *n* (%)	No *n* (%)	Yes *n* (%)	*χ*^2^	*P*-value
Is Bat contaminated the water	18 (9)	74 (37)	108 (54)	26.9	< 0.01
Is bats destroy our environment	23 (11.5)	85 (42.5)	92 (46)	30.2	< 0.01
Do you know zoonotic diseases caused by bats	41 (20)	69 (34.5)	90 (45)	52.5	< 0.01
Is bats creating noise in the environment	10 (5)	84 (42)	106 (5)	15.8	0.01
Did you know about bats help in insects control	20 (10)	61 (30.5)	119 (59.5)	35.7	< 0.01
Do you economical loss inflicted by bats	28 (14)	76 (38)	96 (48)	33.3	< 0.01
Do you know bats feed on pests of crop	27 (13.5)	79 (39.5)	94 (47)	40.5	< 0.01
Is bats are useful in tree planting	33 (16.5)	79 (39.5)	88 (44)	48.4	< 0.01
Can we use bats droppings as fertilizer	26 (13)	77 (38.5)	97 (48.5)	41.1	< 0.01
Have you ever observe bats make fruit drop from tree	14 (7)	59 (29.5)	127 (63.5)	38.6	< 0.01
Is bats help in seeds disperse in environment	20 (10)	68 (34)	112 (56)	35.3	< 0.01
Have you seen any benefit of bats to people	30 (15)	82 (41)	88 (44)	42.2	< 0.01

### Perception and attitude of people

Respondents were asked questions regarding the perception and attitude towards bats (Table 3). An equal number of respondents claimed being friendly towards or against bats (*n* = 92; 46%). The majority of respondents claimed about felt tense in the presence of a bat (*n* = 104; 52%) and declined that the bats bring good luck (*n* = 98; 49%). Among the respondents, a greater number claimed that bats are not demonic (*n* = 100; 50%), although 31% believe this claim. Most of the respondents associated with fruit orchards claimed that they do not kill bats (*n* = 136; 68%), although ~ 30% (*n* = 59) have in the past, and almost all 191 (95.5%) respondents refused killing bats when having an opportunity.

**Table 3:** Respondents count (*n*) and frequency (%) of the responses for questions regarding the perception and attitude of people towards bats

Perception and attitude of people towards bats	Do not know (%)	No (%)	Yes (%)	*χ*^2^	*P*-value
Unfriendly towards bats	16 (8)	92 (46)	92 (46)	22.1	< 0.01
Feel tensed when I see bats	9 (4.5)	104 (52)	87 (43.5)	14.3	0.02
Bats bring good luck	20 (10)	98 (49)	82 (41)	31.9	< 0.01
Bats are demonic	37 (18.5)	100 (50)	63 (31.5)	60.1	< 0.01
I have ever killed bats	5 (2.5)	136 (68)	59 (29.5)	48.1	< 0.01
I will kill bat anytime I have the opportunity	6 (3)	191 (95.5)	3 (1.5)	15.6	< 0.01

### Perception about bats control

Respondents were asked questions regarding the perception about the control of bats (Table 4). Among the respondents, 67 (33.5%) claimed that shooting bats was an appropriate control measure and trees should be cut down to get rid of bats (*n* = 68; 34%). Another 47 (23.5%) respondents claimed that poison should be used to control bats. A higher number of respondents claimed that the bats should be left alone (*n* = 130; 65%) and 45 (22.5%) strongly agreed with this statement.

**Table 4:** Respondents count (*n*) and frequency (%) of the responses for questions regarding the perception about control of bats

Perception about control of bats	Agreed (%)	Disagreed (%)	Do not know (%)	*χ*^2^	*P*-value
Shooting bats with firearms is appropriate	68 (34)	127 (53.5)	5 (2.5)	0.284	0.967
Tree should be cut down to get rid of the bats	74 (37)	124 (62)	2 (1)	0.322	0.851
These bats should be left alone	130 (65)	51 (25.5)	19 (9.5)	2.198	0.333
Poisons should be used	47 (23.5)	108 (54)	45 (22.5)	0.027	0.986

### Non-lethal methods for bats control

**Fig. 2:**
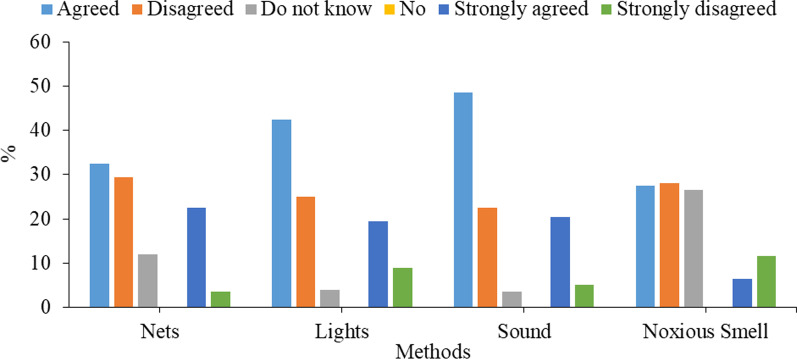
Respondents count (*n*) and frequency (%) of the responses for questions regarding the non-lethal methods usage to control bats

Respondents were asked questions regarding the non-lethal methods adopted to control bats (Fig. 2). A majority of the respondents claimed that nets (*n* = 110; 55%), lights (*n* = 124; 62%), sound (*n* = 138; 69%) and noxious smell (*n* = 68; 34%) should be used to control bats but, a significant number of respondents disregarded the use of nets (*n* = 66; 33%), lights (*n* = 68; 34%), sound (*n* = 55; 27.5%) and noxious smell (*n* = 79; 39.5%) to control the bats.

### Myths about bats

**Fig. 3:**
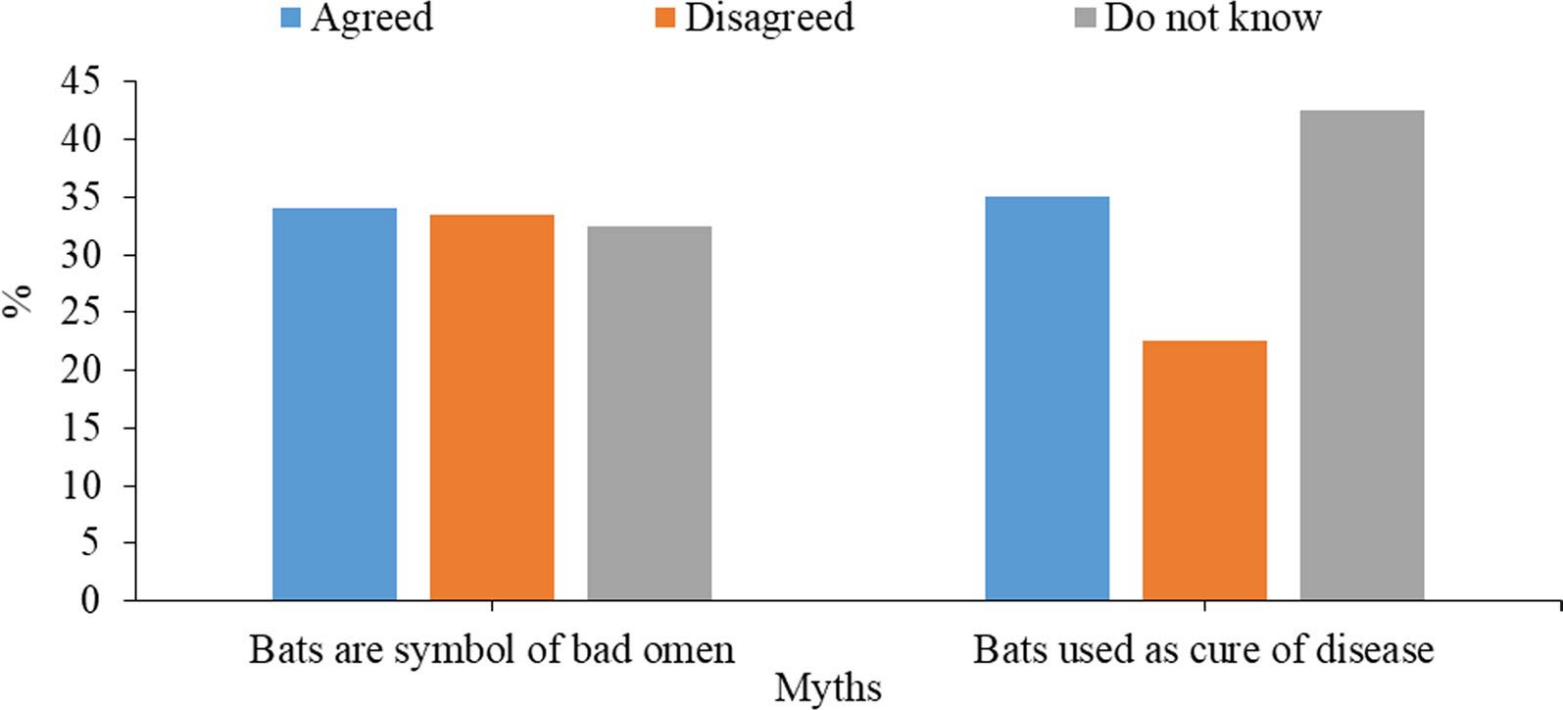
Respondents count (*n*) and frequency (%) of the responses of the questions regarding the myths about bats

Respondents were asked questions regarding myths about bats (Fig. 3). A majority of the respondents either agreed (34%), disagreed (33.50%) or do not know (32.50%) that bats are a symbol of bad omen. The highest number of respondents did not know that bats or bat parts could be used to cure certain diseases (*n* = 85; 42.50%), but a similar (35%) number are agreed and (22.50%) disagreed with this.

## Discussion

This study was carried out in areas surrounded by different fruit orchards, including persimmon, citrus, loquat, litchi, orange, pears, plum, guava, mango and papaya. The majority of the residents were owners of the orchards, labourers working in the orchards and local people associated with these agricultural communities. While we attempted to get a representative sample of the community through our participant enrollment, people of Malakand district did not allow males to interview females due to religious ethics, which was not the case in Sheikhupura district. In a previous study [10] a similar trend was reported where the females were fewer respondents in districts Mardan, Peshawar, and Charsadda, while significant respondents were farmers. In future efforts we will increase our sample size and include female researchers to administer the questionnaire to get a more balanced demographic representation from these communities. Despite this demographic skew, our interviews identified some important factors to consider to promote bat conservation and reduce negative bat-human interactions.

Although bats are very common animals in rural and urban human settlements of Pakistan and frequently encountered in old buildings, orchards, animal sheds and open tree roosts, we found that basic knowledge about bats was lacking. This included an overall misunderstanding of bat taxonomy, as most of the respondents considered bats to be birds and not mammals. The possible reason for this misconception is that the majority of the respondents had a low level of scientific education such as primary education and they considered it as a bird because of its flight mode. Our results are in agreement with previous observations from a survey which was conducted in Bangladesh, where 33.7% participants considered bats as birds [22].

Bats include a diverse array of species, at least 50 species in Pakistan, including the vast majority of which are insectivorous. However, we found that orchard owners, laborers and local peoples consider that bats feed only on fruits. This could be because of their poor observation and lack of basic knowledge about bat biology and feeding behaviors of different groups of bats. Previous studies conducted in Argentina and Slovakia indicated highly negative perceptions of local people regarding the bats feeding behavior, such as feeding on blood of humans and animals, damaging the crops and consuming fruits without any knowledge of bat species and their feeding preferences [19, 23]. In Kenya, Musila et al. [21] reported that *R. aegyptiacus* is a common fruit bat which acts as a pest because this bat species will feed on mangoes, which are a source income for the local residents, and economic losses caused by fruit bats by damaging fruits indirectly influence the livelihood of local residents.

Our study was conducted in areas surrounded by different types of fruit orchards, and according to the fruit farmers and local people, bats were believed to contaminate and directly damage a wide variety of fruits. The increased utilization of agricultural lands by humans for residential purposes may have resulted in a lower availability of natural food for bats thereby increasing fruit orchards visitation and damage [24]. Unfortunately, this human-wildlife conflict has resulted in farmers shooting bats to avoid fruit damage and to reduce their economic losses [10, 25]. In our survey we found that 33% of the participants agreed or strongly agreed that shooting bats was an appropriate control measure. In Mauritius 20,000 fruit bats (*Pteropus niger*) were culled to minimize the losses to mango and lychee [25]. Similar indiscriminate culling was documented by Mahmood ul Hassan et al. [10], in districts Peshawar and Charsadda of KPK province of Pakistan where more than 200 bats were killed by a single person.

Almost half of our respondents 46% exhibited an unfriendly attitude towards bats and agreed they would try to kill bats as soon as they would use them. Such an attitude is probably linked to species biases towards colorful animals and birds with negative feelings towards cryptic or less attractive animals such as bats, rats, invertebrates and reptiles [26]. Forty-three percent of the people stated they become tense when they see bats and 52% of them said that seeing bats had an impact on their psychic condition. Half of our respondents believe that bats are demonic and have negative impacts on human lives.

Persecution against fruit bats is not unique to Pakistan. A study conducted in Malaysia found that 79% of local people ostracized flying foxes, because of their noise, smell from faces and damage to fruit trees [27]. Moreover, negative local responses like raiding of fruit-crop has also been found in the Cook Islands [28] and Kenya [21]. Farmers in Puebla, Mexico dislike bats living in their farms and 16 percent (*n* = 36) of those participating in the survey said they kill the bats when found inside their houses [29]. Additionally, in Argentina 14% people kill bats when bats enter in the home, office and farms [30]. While Reid [31] reported that Costa Rican men (27%) kill bats on their farms. We found that a high number of respondents claimed that bats inflict economical losses (*n* = 96; 48%) by dropping fruits from trees (*n* = 127; 63.5%), therefore, bats have no benefit to people (*n* = 88; 44%). While there is some evidence that economic losses to orchards are associated with bats [32–35], other studies, i.e. Korine et al. [36] have shown that the impact of bats on agriculture and economic losses have been exaggerated. Furthermore, there are several non-lethal mitigation techniques available [27].

We found a high percentage of people strongly agreed towards the control of bats by using non-lethal methods including netting, lights and sound. Netting (of a type that avoids bat entanglement) is an effective non-lethal method to protect orchards from bats and other wild fauna, as demonstrated by studies in Australia [37]. Netting has been used to control commercial crops from bats in Israel [36]. While another study conducted in Madagascar on commercial fruits of the lychee tree (*Litchi chinensis Sonn*.) by using three methods, such as flags, ring bell and unpleasant smell. Between these three methods plastic flags and bell ringing were less effective in reducing the fruit bat damage compared with the taste deterrent [38]. Another method involves harvesting unripe fruits several days before they become attractive to bats [39, 40]. In Malaysia the most common method used (23% of respondents) to remove the bats was to light fires under trees to smoke them out [41]—but this is an invasive method that only serves to stress the animals. However, there are still challenges associated with netting to deployment in Australia, which has resulted in a low acceptance among some growers [42, 43]. Netting is also unsuitable for banana orchards and hilly area plantations, as it is impossible to cover with netting [44–46].

In our study questionnaires were circulated to find out the views of people about bad and good omens about bats. About 34% of the respondents strongly disagreed with the myth that the bats are related with bad omens. In contrast to our study, a study conducted in India reported 36.5% and 54.5% good and bad omens, respectively, associated with bats [47]. Another study reported 96.1% people showed bad omens in Peshawar and 63.1% in Charsadda have responded that bats are an emblem of bad omens [10]. While we found several lines of evidence for a negative perception of bats in our study, 47% of people thought that bats are useful for the control of insects which act as pests for their fruits and acknowledged that bats provide other ecosystem services (seed dispersal and use of bat guano for fertilizer). Similarly, we observed positive sentiment about bats as 41% of respondents believe in the myth that the bats bring good fortune into their houses and business. A previous study was conducted on the island of Rodrigues (Republic of Mauritius) and observed that 13% of the respondents who accepted cultural myths and believe that ‘bats bring good luck [48]. Since the fourteenth century, Chinese culture has associated bats with good luck and blessings [49]. These lucky bat symbols were prevalent in Chinese art throughout the centuries, but this symbolic concept struggles to find its place in the larger global narratives about bats today, and bat symbolism in other Asian cultures, including in Pakistan, remains largely unknown. We observed the potential for broader support for bat conservation from our relatively limited sampling of the community. This includes 54% of participants who were against killing bats by poison (62%) and a high percentage of people in favor of not cutting down trees where bats were residing.

Bats have a habit of partially eating the fruits and fruit contamination by their urine and saliva has previously been linked to public health concerns [50, 51]. In our study 41.5% of people think that bats are the source of spreading infectious diseases. This finding was similar with Castilla et al. [30] from Argentina, where 42% of local peoples and farmers had a concept that bats transmit diseases. In Bangladesh, it was concluded that people have inadequate knowledge of bats and their role in the spread of the Nipah virus [22]. Another study was conducted in Bangladesh for determining the risk assessment for variation in geographical variation of Nipah virus infection due to ecological factors and human behavior. It was concluded that numbers of persons, bats, and consumption of raw date palm sap was associated with the spread of said the virus [51]. Local community training and education about zoonotic diseases, especially those transmitted by bats is very important to prevent bat-borne disease outbreaks or pandemic [52]. A high correlation was found between lack of knowledge and spread of zoonotic disease in Nigeria [53]. Because of this lack of information, seeking health advice when exposed to bats may be difficult [54, 55]. One way to prevent possible indirect transmission of pathogens from fruit bats is to wash fruit prior to consumption and avoid fruit partially eaten by bats. According to our study most of the people (97%) wash fruits before consumption. This is a very high rate as compared with 52% of people surveyed in West Africa, who wash the fruit before consumption [56]. The high proportion of people in Pakistan who wash fruits could have additional value in preventing bat to human pathogen transmission, even though the habit of washing fruits by people is because of their perception about dust and high levels of insecticides sprays on fruits, as well as rich diversity of bats surrounding orchards.

In Pakistan, bat consumption is typically viewed as rare or absent because of religious and ethical issues. Similar findings were observed in Malaysia from people living on Tioman Island, where people do not eat bats because of religious customs. However, the trading of flying foxes was at a peak in this region twenty years ago for the consumption by the Chinese population [41]. This differs from other parts of the world, for example in Thailand, where 42% of the respondents used bats as food [57]. Interestingly, one third of the participants in our study (31%) agree that bats can be used therapeutically. A related study found that local people of Bangladeshi (17%) used bats for medicine [58]. In Pakistan bats are hunted by local “Hakeems” (local health practitioners) for their body fat to be used as a potion and as a cure for rheumatic pains [20]. Similar responses were noted in a previous study, in which eight respondents in Peshawar (6.6%) district and 17.5% respondents in Charsadda district (*n* = 18) were of the view that bats are used either as aphrodisiacs or for curing baldness [10]. In India bats such as *Cynopterus sphinx* are used in roasted form among the peoples of the Tangsa tribe to control bed-wetting in children and liver problems in adults [59]. While the meat and bones of fruit bats are used to cure hepatitis and to treat mental illness among Gamo Gofa tribals of Ethiopia [60].

## Conclusions

This survey has provided us with information on the level of knowledge by residents of Punjab and the KPK province in regard to bats. Moreover, their perception and attitudes were evaluated. Views on bats varied amongst the respondents and depended on the latter’s gender, age, level of education and occupation. Pakistan has a diverse fauna of bats and the present study can be used to improve the knowledge of the local people about bats, their ecological role and how we can live safely with bats to mitigate crop damage and the emergence of zoonotic diseases. National level workshops and seminars could help improve awareness of the local community especially for orchard owners to minimize conflicts in the region between humans and bats. Early childhood education modules that include basic information about bat ecology and biology would improve the general knowledge about these important animals in the local communities. Our study provides preliminary data to help build on in additional research and in pilot programmes to develop future policies for the conservation of local bats species.

## Data Availability

All data generated or analyzed during this study are included in this published article and its supplementary information files.
